# Drug-coated balloon angioplasty for symptomatic vertebral artery origin stenosis: a real-world analysis of efficacy and predictors for bailout stenting

**DOI:** 10.3389/fneur.2026.1804766

**Published:** 2026-04-14

**Authors:** Yuerong Dong, Dong Zhang, Yan Zhang, Qing He

**Affiliations:** Department of Neurology, The Affiliated Xuzhou Municipal Hospital of Xuzhou Medical University, Xuzhou, Jiangsu, China

**Keywords:** angioplasty, bailout stenting, drug-coated balloon, predictive factors, vertebral artery origin stenosis

## Abstract

**Objective:**

This study aims to evaluate the preliminary efficacy and safety of drug-coated balloon (DCB) angioplasty for treating symptomatic vertebral artery origin stenosis (VAOS) in a real-world setting and to investigate potential factors necessitating intraoperative bailout stenting (BS).

**Methods:**

This single-center retrospective study enrolled 121 consecutive symptomatic VAOS patients treated with DCB angioplasty at Xuzhou First People’s Hospital between January 2022 and December 2024. Patients were classified into the DCB group or the BS group based on the requirement for intraoperative bailout stenting. Stenosis rates were compared within the DCB group at baseline, immediately post-procedure, and at ≥3- and ≥12-month follow-up, as well as between the DCB and BS groups at corresponding time points. The least absolute shrinkage and selection operator (LASSO) regression and multivariable logistic regression were used for variable selection, and a nomogram was constructed and evaluated using the concordance index (*C*-index) and the Hosmer–Lemeshow goodness-of-fit test.

**Results:**

Technical success was achieved in 94 patients (77.6%), while bailout stenting was required in 27 patients (22.3%). The median stenosis of the DCB group decreased from 83% at baseline to 36% immediately post-procedure (*p* < 0.001) and remained stable at ≥3 months (43%) and ≥12 months (45%). At ≥3 months, stenosis was lower in the DCB group than in the BS group (40.5% vs. 47%, *p* < 0.05), whereas no significant difference was observed at ≥12 months (48% vs. 69%, *p* = 0.17). Multivariable analysis identified elevated diastolic blood pressure [odds ratio (OR) = 1.051, 95% CI: 1.011–1.093] and concomitant internal carotid artery stenosis (moderate stenosis: OR = 8.377, 95% CI: 1.977–35.493; severe stenosis or occlusion: OR = 6.346, 95% CI: 1.493–26.975) as independent predictors of bailout stenting. The nomogram demonstrated good discrimination (*C*-index = 0.807, 95% CI: 0.709–0.906) and adequate calibration (*p* = 0.9158).

**Conclusion:**

DCB angioplasty was associated with favorable angiographic durability in symptomatic VAOS, with similar long-term patency between balloon-only and bailout stent strategies. Higher diastolic blood pressure, and concomitant carotid stenosis were associated with bailout stenting.

## 1 Introduction

Vertebral artery origin stenosis (VAOS) is a major contributor to posterior circulation ischemic stroke and transient ischemic attack, accounting for up to 20% of posterior circulation events (1). Due to its unique anatomical location and complex hemodynamic characteristics, the vertebral artery origin is one of the most susceptible sites for atherosclerosis (2). Although current guidelines recommend intensive medical management as first-line therapy, endovascular revascularization remains an important therapeutic option for patients with recurrent or refractory symptoms (3–6).

Conventional endovascular approaches, particularly plain balloon angioplasty and bare-metal stent (BMS) implantation, are limited by high restenosis rates caused by vascular elastic recoil and intimal hyperplasia (7, 8). Drug-eluting stents (DES) have been shown to reduce restenosis, with reported rates of 18.4% compared with 31.1% for BMS (9). However, DES are associated with risks such as stent thrombosis, stent fracture, and long-term complications related to permanent implantation (10, 11).

Drug-coated balloon (DCB) angioplasty has emerged as a “leave nothing behind” strategy that delivers antiproliferative agents locally to inhibit intimal hyperplasia without permanent implantation (12). DCB has demonstrated favorable safety and restenosis profiles in coronary and peripheral artery disease (13–16). In the cerebrovascular field, early pilot studies and small observational cohorts suggested that DCB angioplasty may be feasible and safe for VAOS, with encouraging angiographic outcomes (17–22). Recently, the first multicenter randomized controlled trial demonstrated that DCB significantly reduced 12-month restenosis compared with BMS (13.2% vs. 38.5%) while maintaining similar periprocedural and 1-year safety outcomes (23).

Despite these promising findings, there is still a lack of large real-world DCB-treated VAOS cohorts and relatively high rates of intraprocedural bailout stenting (BS), reaching 22.2% in DCB-VAOS trial. The factors contributing to BS in VAOS have not been examined, and the prognostic outcomes of patients undergoing BS compared with those treated with DCB alone remain unclear.

Accordingly, this study reports a real-world retrospective cohort of symptomatic severe VAOS patients to evaluate the efficacy and safety of DCB and explore clinical predictors of BS using multivariable modeling. As the largest reported DCB-treated VAOS cohorts to date, this study aims to complement emerging randomized evidence by providing real-world insights into procedural outcomes and risk stratification.

## 2 Subjects and methods

### 2.1 General information

A retrospective analysis was conducted on a consecutive series of 121 patients with symptomatic VAOS who underwent paclitaxel-coated DCB (P-DCB) angioplasty at Xuzhou First People’s Hospital between January 2022 and December 2024. The requirement for informed consent was waived due to the retrospective nature of the study. This study was approved by the Medical Ethics Committee of Xuzhou First People’s Hospital (Approval No. xyyll[2025]184) and adhered to the ethical principles of the Declaration of Helsinki.

### 2.2 Inclusion and exclusion criteria

The inclusion criteria were as follows: (1) Age 18–80 years; (2) After optimal medical treatment, patients who experienced posterior circulation ischemic events with culprit vessel stenosis ≥70%, or culprit vessel stenosis ≥60% with newly confirmed infarction lesions consistent with the stenotic site on imaging, and excluding other cardiogenic embolic factors; (3) modified Rankin Scale (mRS) score ≤3; (4) agreement to undergo percutaneous endovascular treatment (including bare-metal stent implantation, DCB angioplasty).

The exclusion criteria were as follows: (1) mild vertebral artery origin stenosis (< 50%) without clinical symptoms; (2) coagulation disorders or contraindications to antiplatelet or anticoagulant therapy; (3) severe cardiac, pulmonary, hepatic, or renal dysfunction that prevented tolerance to the procedure; (4) history of intracranial hemorrhage within the last 3 months or the presence of large intracranial aneurysms, complex arteriovenous malformations, arteriovenous fistulas, or other conditions associated with a high risk of hemorrhage.(5) The patients with cerebral infarction whose infarction area extends to one-third or more of the posterior circulation territory.

**Figure 1:**
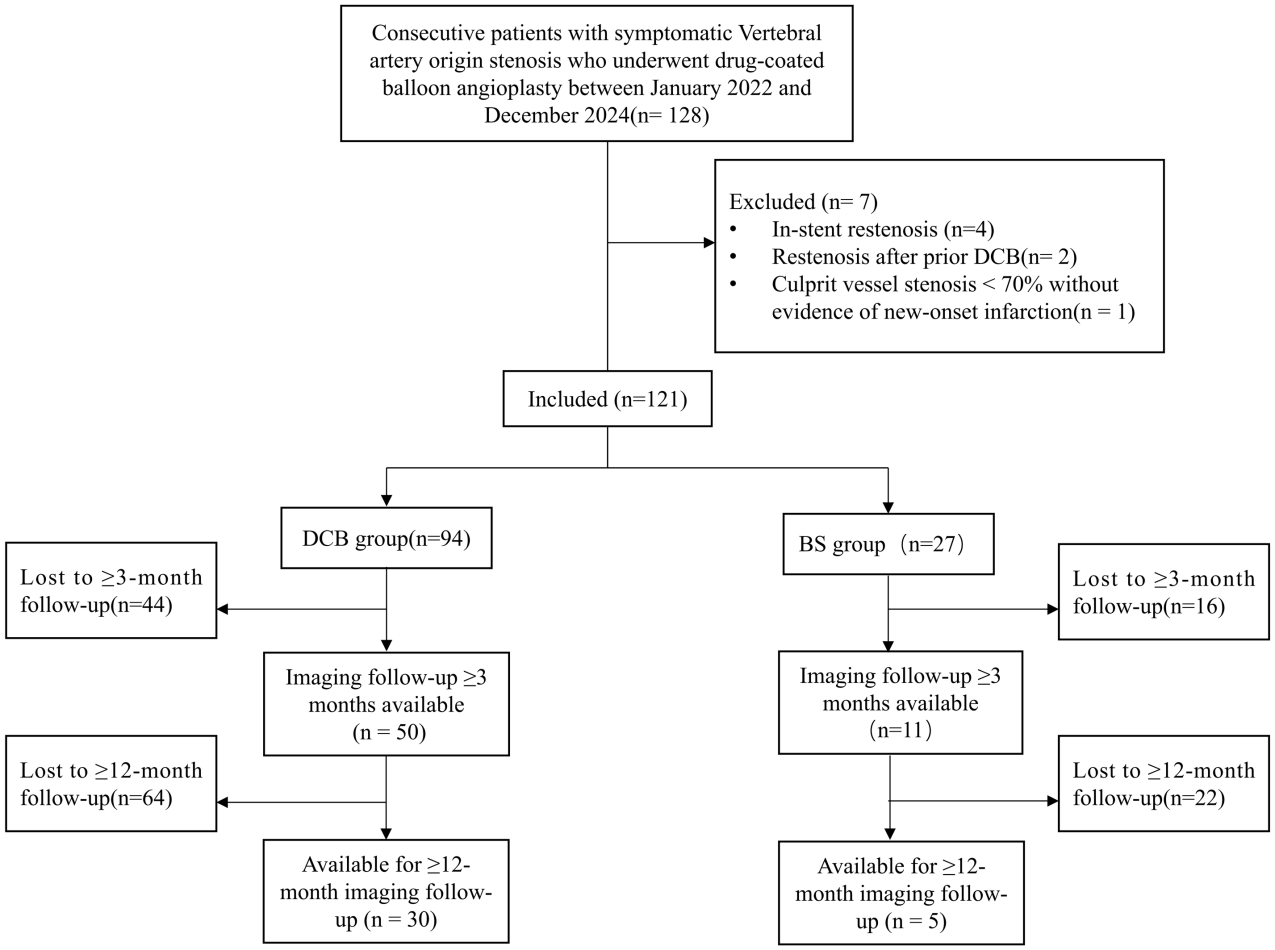
Flowchart of patient selection and study enrollment. DCB, drug-coated balloon; BS, bailout stenting.

The comprehensive screening and enrollment process is listed in the flow chart (Figure 1).

### 2.3 Treatment protocol

#### 2.3.1 Preoperative preparation

Upon admission, all patients underwent routine hematological tests, including complete blood count, liver and kidney function tests, coagulation profile, and D-dimer level. Relevant imaging studies, specifically computed tomography angiography (CTA) of the head and neck and digital subtraction angiography (DSA), were performed. To ensure data consistency, all CTA examinations were conducted using the same dual-source CT scanner (Siemens Healthcare, Forchheim, Germany) and reviewed by a dedicated team of experienced radiologists. All endovascular procedures were performed by the same interventionists with more than 10 years of experience. Patient comorbidities were fully assessed, vascular evaluation was completed, and patient selection was guided by cerebral perfusion status when necessary. Collateral circulation and vascular reserve capacity were thoroughly evaluated to exclude patients who were unsuitable for endovascular therapy. All patients received standard dual antiplatelet therapy (aspirin 100 mg and clopidogrel 75 mg daily) for at least 5 days prior to the procedure. Platelet reactivity was assessed using thrombo-elastography (TEG), and the procedure was performed only when the platelet inhibition rate exceeded 50%. The results were interpreted by laboratory personnel according to the institutional testing protocol. If inadequate platelet inhibition was detected, clopidogrel was replaced with alternative ticagrelor (90 mg twice daily) until an adequate antiplatelet response was achieved.

#### 2.3.2 Operative procedure

All procedures were performed using a vascular angiography system (Artis Zee Ceiling; Siemens). Intravenous heparin (0.5–0.6 mg/kg) was administered to maintain the activated clotting time (ACT) between 250 and 300 s, with an additional half dose given if necessary after 1 h. The reference vessel diameter (RVD) was measured using 2D quantitative vascular angiography (QVA) on the diagnostic workstation. Intravascular ultrasound (IVUS) was not utilized in this study, as the device had not yet been approved for cerebrovascular applications at our institution. Under local anesthesia, the femoral artery was punctured, and an 8 French arterial sheath was introduced (a 6 French sheath was used for radial artery access). An 8F guiding catheter was typically used to establish vascular access, and the optimal working projection angle was selected. Under roadmap guidance, a 0.014-inch microguidewire was navigated across the origin of the vertebral artery and advanced to the distal V1 segment. An embolic protection device (Spider FX Embolic Protection Device, ev3) was then delivered and deployed in the distal V1 segment. A paclitaxel-coated balloon (AcoArt Dahlia; Beijing Acotec Scientific Co., Ltd.) sized to match the vessel diameter was selected. Cerebral angiography was then performed to assess the degree of stenosis. Currently, there is no consensus regarding the routine use of predilatation in vertebral artery origin stenosis (22). In our center, predilatation was not routinely performed to minimize mechanical injury to the vertebral artery ostium and to reduce the risk of dissection caused by repeated balloon inflation. Predilatation was reserved only for cases in which the drug-coated balloon (DCB) could not cross the stenotic lesion. In such cases, a 2.0 × 15 mm or 3.0 × 15 mm uncoated balloon was used for gentle predilatation to facilitate device delivery. A strict balloon-to-vessel diameter ratio of 0.8–1.0 was applied to minimize the risk of dissection caused by over-sizing. Paclitaxel-coated balloon sizes used ranged from 4 × 30 mm to 5 × 30 mm. The balloon was inflated slowly to the nominal pressure (6–14 bar) until the waist indentation disappeared, and then maintained for a target time of 180 s. After slow deflation, post-intervention angiography was performed to measure residual stenosis, assess changes in posterior circulation flow, and rule out adverse events, such as vessel rupture, distal embolism, or access-site hematoma. If significant elastic recoil (>50%) persisted, a prolonged inflation or repeated dilation was performed. Follow-up cerebral angiography was performed to reassess the vascular stenosis and cerebral perfusion. After satisfactory results were achieved, the protective device and balloon were removed. Bailout stenting (BS) with a bare-metal stent (Herkulink, 4 × 18 mm or 4 × 15 mm) was performed immediately only under the following criteria: (1) flow-limiting dissection [National Heart, Lung, and Blood Institute (NHLBI) type C or higher]; or (2) severe elastic recoil resulting in >50% residual stenosis or flow compromise despite repeated dilation.

#### 2.3.3 Postoperative management

Patients underwent routine cardiac and neurological monitoring. Observations included bleeding at the puncture site, palpation of the dorsalis pedis artery pulses, and neurological symptoms. All patients took a combination of clopidogrel (75 mg/day) and aspirin (100 mg/day) for 3 months after the procedure, followed by long-term monotherapy with either clopidogrel or aspirin. Standard postoperative management also included lipid-lowering therapy, anticoagulation when indicated, and risk factor control.

### 2.4 Data collection

The epidemiological, clinical, and imaging data of all patients were obtained from the medical records. Demographic information (age, sex, height, and weight), vascular risk factors (hypertension, diabetes mellitus, dyslipidemia, history of heart disease, atrial fibrillation, smoking and alcohol history, and presence of other intracranial large-vessel stenosis), laboratory results, and preoperative clinical functional status (mRS score) were collected from all enrolled patients. Procedural data, including lesion location, preoperative stenosis rate, immediate postoperative stenosis rate, perioperative complications (such as vessel dissection, rupture, and distal embolism), and the need for BS, were also recorded. Postoperative mRS scores were obtained through outpatient reviews or telephone follow-ups. Stenosis rates at a minimum of 3 and 12 months postoperatively were assessed using follow-up CTA.

### 2.5 Primary endpoints and definitions

#### 2.5.1 Technical success

Defined as successful DCB dilation of the lesion, resulting in a residual stenosis rate of < 50% post-procedure, without any severe complications.

#### 2.5.2 Stenosis rate measurement

VAOS degree was assessed in all patients using DSA. Postoperative and follow-up assessments of lumen patency and restenosis in the treated vessels were performed using CTA of the head and neck or DSA. The North American Symptomatic Carotid Endarterectomy Trial criteria (24) were applied as follows:


$$\begin{array}{l} \text{The stenosis rate}\\ =(\begin{array}{l} 1-\text{Luminal diameter}\;\mathrm{at}\;\text{the narrowest point}\\ /\text{Luminal diameter of the distal normal vessel} \end{array})\times 100\%. \end{array}$$

#### 2.5.3 Restenosis

Defined as a stenosis rate > 50% at the target lesion on follow-up imaging (≥ 3 months post-procedure).

#### 2.5.4 Safety endpoint

Any procedure-related complication occurring in the perioperative period (intraoperative to within 30 days post-procedure), including death, stroke, vessel dissection, or vessel rupture.

### 2.6 Statistical analysis

The Statistical Package for Social Sciences (version 26.0) and R Studio (version 4.2.1) software were used for statistical analyses. Data are presented as mean ± standard deviation, percentages, or interquartile range (IQR) according to the data type. The Shapiro–Wilk test was used to assess the normality of the data. The Wilcoxon signed-rank test was used to compare non-normally distributed data, and the paired-samples *t*-test was used for normally distributed data.

Using R Studio (version 4.2.1) and the glmnet package (version 4.1.7), least absolute shrinkage and selection operator (LASSO) regression was applied to the cleaned data to obtain variable coefficients, log(lambda) values, and L1 norm values, with visualization of the analysis data. The optimal lambda value for LASSO regression was selected using 10-fold cross-validation. Predictors of BS identified from screening were included in the multivariate logistic regression analysis.

Multicollinearity was assessed using the variance inflation factor (VIF). Variables with a final *p*-value <0.05 were considered significant, and a forest plot of relevant variables was generated using the ggstatsplot package. A nomogram was constructed based on the logistic regression results. The discriminative ability of the model was evaluated using the area under the receiver operating characteristic (ROC) curve (*C*-index). Model calibration was assessed using the Hosmer–Lemeshow goodness-of-fit test.

To assess potential attrition bias resulting from incomplete imaging follow-up, baseline clinical and anatomical characteristics were compared between patients with and without angiographic follow-up at both the ≥3-month and ≥12-month time points. In addition, given the substantial proportion of missing follow-up imaging data, a sensitivity analysis was performed to estimate restenosis rates under two extreme assumptions: best-case scenario (assuming no restenosis in patients lost to follow-up) and worst-case scenario (assuming restenosis occurred in all patients lost to follow-up).

All statistical tests were two-tailed, and *p* < 0.05 was considered statistically significant.

## 3 Results

### 3.1 Patient baseline characteristics

A total of 121 patients were enrolled in this study. Based on intraoperative outcomes, patients were categorized into DCB (*n* = 94, 77.69%) and BS (*n* = 27, 22.31%) groups. The baseline clinical and angiographic characteristics of the two groups are summarized in Table 1.

**Table 1:** Comparison of baseline clinical and angiographic characteristics of patients undergoing DCB angioplasty with or without bailout stenting for vertebral artery origin stenosis.

Variables	Total (*n* = 121)	DCB group (*n* = 94)	BS group (*n* = 27)	*p* Value
Age, year (M, IQR)	70.00 (61.00, 75.00)	71.00 (61.00, 75.00)	67.00 (61.50, 73.00)	0.251^b^
Male, *n* (%)	99 (81.82)	77 (81.91)	22 (81.48)	0.964^c^
BMI, Kg/m^2^ (m ± s)	24.09 ± 3.08	24.28 ± 2.92	23.40 ± 3.55	0.191^d^
Admission SBP, mmHg (m ± s)	141.16 ± 21.70	139.55 ± 20.99	146.74 ± 23.58	0.130^d^
Admission DBP, mmHg (m ± s)	82.00 ± 13.19	80.22 ± 12.60	88.19 ± 13.58	0.005^a^^,^^d^
Fasting blood glucose, mmol/L, (M, IQR)	5.70 (5.00–7.20)	5.72 (5.03–7.23)	5.60 (4.73–6.85)	0.440^b^
Triglycerides level, mmol/L (M, IQR)	1.27 (0.92, 1.85)	1.29 (0.92, 1.88)	1.24 (0.88, 1.58)	0.478^b^
Total cholesterol, mmol/L (M, IQR)	3.61 (2.98, 4.28)	3.59 (3.00, 4.20)	3.76 (2.99, 4.73)	0.674^b^
LDL cholesterol level, mmol/L (M, IQR)	2.31 (1.85, 2.88)	2.31 (1.91, 2.84)	2.60(1.83, 3.08)	0.375^b^
Hypertension, *n* (%)	89 (73.55)	73 (77.66)	16 (59.26)	0.056^c^
Diabetes mellitus, *n* (%)	48 (39.67)	35 (37.23)	13 (48.15)	0.307^c^
Hyperlipidemia, *n* (%)	9 (7.44)	6 (6.38)	3 (11.11)	0.682^c^
Cardiopathy, *n* (%)	28 (23.14)	22 (23.40)	6 (22.22)	0.898^c^
Atrial fibrillation, *n* (%)	3 (2.48)	2 (2.13)	1 (3.70)	0.535^d^
Current smoking, *n* (%)	17 (14.05)	12 (12.77)	5 (18.52)	0.657^c^
Alcohol consumption, *n* (%)	7 (5.79)	6 (6.38)	1 (3.70)	0.954^c^
Preoperative stenosis percentage (%, M, IQR)	83 (75, 90)	82 (75, 90)	84 (80, 91)	0.344^b^
Target vessel side, *n* (%)				0.608^c^
Right	71 (58.68)	54 (57.45)	17 (62.96)	
Left	50 (41.32)	40 (42.55)	10 (37.04)	
Dominant, *n* (%)				0.496^c^
Codominant side	49 (40.50)	37 (39.36)	12 (44.44)	
Dominant side	56 (46.28)	46 (48.94)	10 (37.04)	
Non-dominant side	16 (13.22)	11 (11.70)	5 (18.52)	
Acute infarction on imaging, *n* (%)	31 (25.62)	27 (28.72)	4 (14.81)	0.145^c^
Internal carotid artery stenosis, *n* (%)				0.004^a^^,^^e^
No	51 (42.15)	47 (50.00)	4 (14.81)	
Mild or moderate	34 (28.10)	22 (23.40)	12 (44.44)	
Severe or total occlusion	36 (29.75)	25 (26.60)	11 (40.74)	
Admission mRS, *n* (%)				0.194^e^
0	7 (5.79)	4 (4.26)	3 (11.11)	
1	95 (78.51)	73 (77.66)	22 (81.48)	
2	19 (15.70)	17 (18.09)	2 (7.41)	

M, median; IQR, interquartile range; DCB, drug-coated balloon; BS, bailout stenting; BMI, body mass index; SBP, systolic blood pressure; DBP, diastolic blood pressure; FBG, fasting blood glucose; LDL, low-density lipoprotein; HDL, high-density lipoprotein.

a *p* < 0.05.

b Mann–Whitney test.

c Chi-square test.

d *t*-test.

e Fisher exact.

No statistically significant differences were observed between the two groups in terms of age, sex, body mass index (BMI), blood lipid levels, diabetes, preoperative stenosis severity, target vessel side, vertebral artery dominance, presence of acute infarction on imaging, admission modified Rankin Scale (mRS) score, or other cardiovascular comorbidities, smoking status, and alcohol consumption (all *p* > 0.05).

Patients in the BS group had significantly higher admission DBP than those in the DCB group (88.19 ± 13.58 vs. 80.22 ± 12.60 mmHg, *p* = 0.005). Moreover, concomitant internal carotid artery stenosis (ICAS) was more frequently observed in the BS group (*p* = 0.004).

### 3.2 Immediate procedural outcomes and efficacy analysis

Technical success was achieved in 94 patients (77.6%), whereas BS was required in 27 patients (22.3%), including 12 cases due to intraoperative dissection and 15 cases due to suboptimal dilation caused by severe elastic recoil.

**Figure 2:**
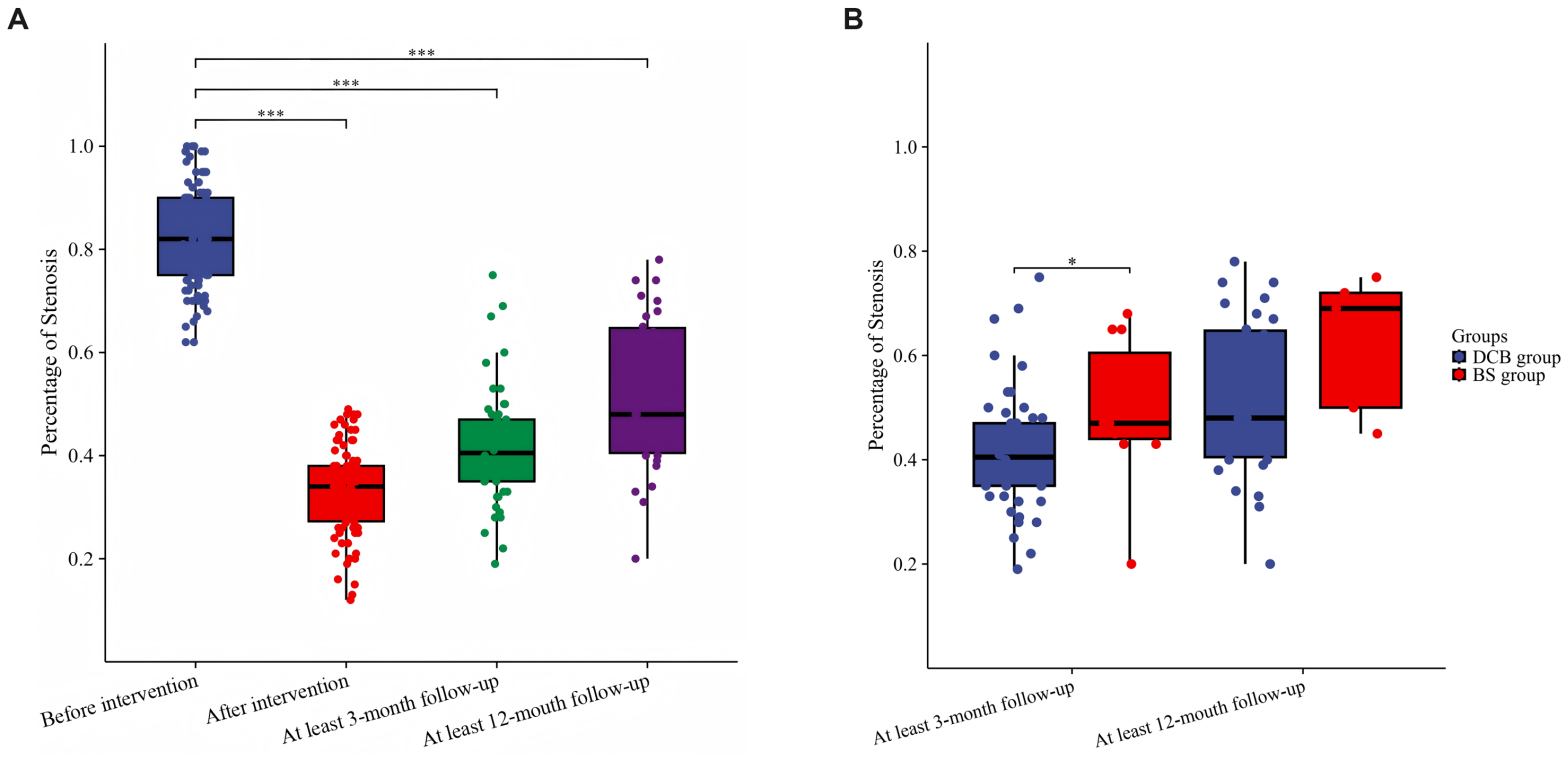
Changes in stenosis severity after drug-coated balloon angioplasty. **(A)** Temporal changes in stenosis in the DCB group. **(B)** Comparison of stenosis rate in the DCB and BS groups at ≥3- and ≥12-month follow-ups.

Overall, the median stenosis rate significantly decreased from 83% (IQR 75–90%) before the procedure to 36% (IQR 25–45%) immediately after treatment (*p* < 0.001) (Figure 2A). Angiographic follow-up was available in 61 patients at ≥3 months (DCB: n = 50; BS: n = 11) and in 35 patients with ≥12-month follow-up (DCB: *n* = 30; BS: *n* = 5) (Figure 2B). Baseline characteristics were compared between patients with and without imaging follow-up to assess potential attrition bias (Supplementary Table S1). No significant differences were observed at ≥12 months. At ≥3 months, a difference in sex distribution was observed, whereas other baseline variables were comparable between groups.

Among patients with follow-up imaging, the median stenosis rate was 43% (IQR 28–50%) at ≥3 months and 45% (IQR: 30–65%) at ≥12 months, both significantly lower than the preprocedural stenosis rate (*p* < 0.001) (Figure 2A). At ≥ 3 months, the median stenosis rate was lower in the DCB group (40.5%) than in the BS group (47%) (*p* < 0.05). Among patients with follow-up beyond 12 months, the median stenosis rate was 48% in the DCB group and 69% in the BS group, although this difference did not reach statistical significance (*p* = 0.17) (Figure 2B).

In the cohort with ≥3-month angiographic follow-up, angiographic restenosis occurred in 7 of 50 patients (14.0%) in the DCB group and 5 of 11 patients (45.5%) in the BS group (*p* = 0.023). To evaluate the potential impact of missing follow-up data, sensitivity analyses were performed under best- and worst-case assumptions (Table 2). The results remained consistent under both assumptions, suggesting that the main findings were robust and unlikely to be substantially affected by follow-up bias.

**Table 2:** Sensitivity analysis of ≥3-month restenosis outcomes according to different assumptions regarding patients without imaging follow-up.

Scenario	DCB restenosis	BS restenosis
Observed (≥3-month imaging follow-up cohort)	7/50 (14.0%)	5/11 (45.5%)
Best-case scenario^a^	7/94 (7.4%)	5/27 (18.5%)
Worst-case scenario^b^	51/94 (54.3%)	21/27 (77.8%)

a Assuming no restenosis in all patients lost to follow-up.

b Assuming restenosis in all patients lost to follow-up.

### 3.3 Safety outcomes

Minor intraoperative vascular dissection occurred in 5 patients (4.1%), none of which resulted in flow limitation or required specific intervention. A total of 27 patients (22.3%) required bailout bare-metal stenting due to either flow-limiting intraoperative dissection (*n* = 12) or suboptimal luminal gain caused by severe elastic recoil (*n* = 15).

No major complications, such as death, major stroke, or vessel rupture, occurred during the perioperative period. During the available clinical follow-up, two patients reported transient ischemic attacks (TIA) in the posterior circulation territory (one in the DCB group and one in the BS group), both cases resolved with medical management (Table 3).

**Table 3:** Clinical outcomes within perioperative period and within 12 months after the procedure.

Follow-up period	Perioperative period		Within 12 months	
	DCB group	BS group	DCB group	BS group
No of patients followed up	94	27	51	11
Vessel rupture	0	0	0	0
TIA	0	0	1	1
Stroke	0	0	0	0
Death	0	0	0	0
mRS score ≥3 in survival	0	0	0	0

DCB, drug-coated balloon; BS, bailout stenting; TIA, transient ischemic attack; mRS, modified Rankin Scale.

### 3.4 Analysis of factors influencing the efficacy of DCB angioplasty

**Figure 3:**
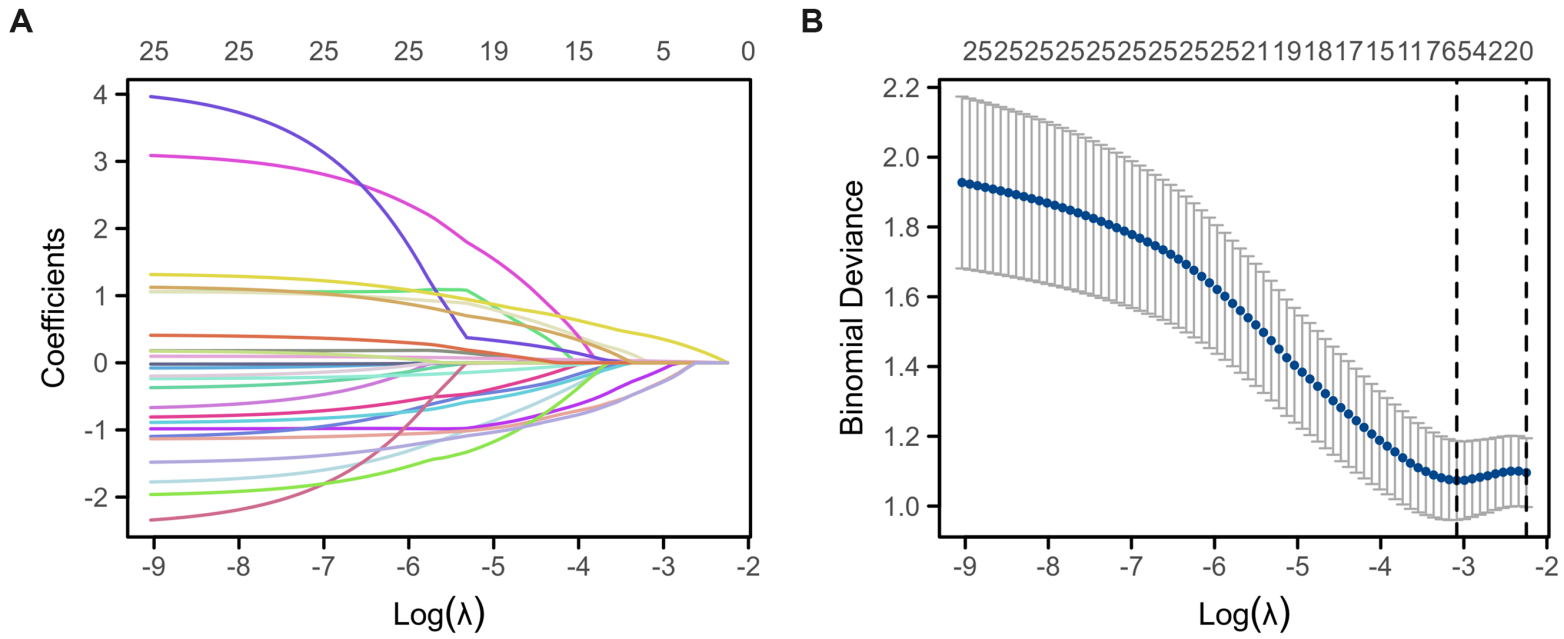
Variable selection using LASSO regression. **(A)** LASSON regression coefficient curve. **(B)** Likelihood deviation graph of predictors.

To mitigate the risk of model overfitting and address potential multicollinearity associated with the high variable-to-event ratio, the least absolute shrinkage and selection operator (LASSO) regression was applied for feature selection. A total of 25 candidate variables were initially included in the LASSO model. As shown in the coefficient profile plot (Figure 3A), the regression coefficients gradually shrank toward zero as the penalty parameter (*λ*) increased.

The optimal value of λ was determined using 10-fold cross-validation based on the minimum binomial deviance (Figure 3B). The dashed vertical line represents the optimal *λ* (*λ*min), corresponding to the model with the best predictive performance. Five variables with non-zero coefficients were ultimately selected: acute infarction, hypertension, admission diastolic blood pressure (DBP), internal carotid artery stenosis, and modified Rankin Scale (mRS) score.

These variables were subsequently entered into the multivariate logistic regression analysis. In addition, several clinically relevant variables, including BMI, admission systolic blood pressure, Dominant, were retained in the model to improve clinical interpretability, while multicollinearity was assessed using the variance inflation factor (VIF). All variables demonstrated VIF values below 8.

**Figure 4:**
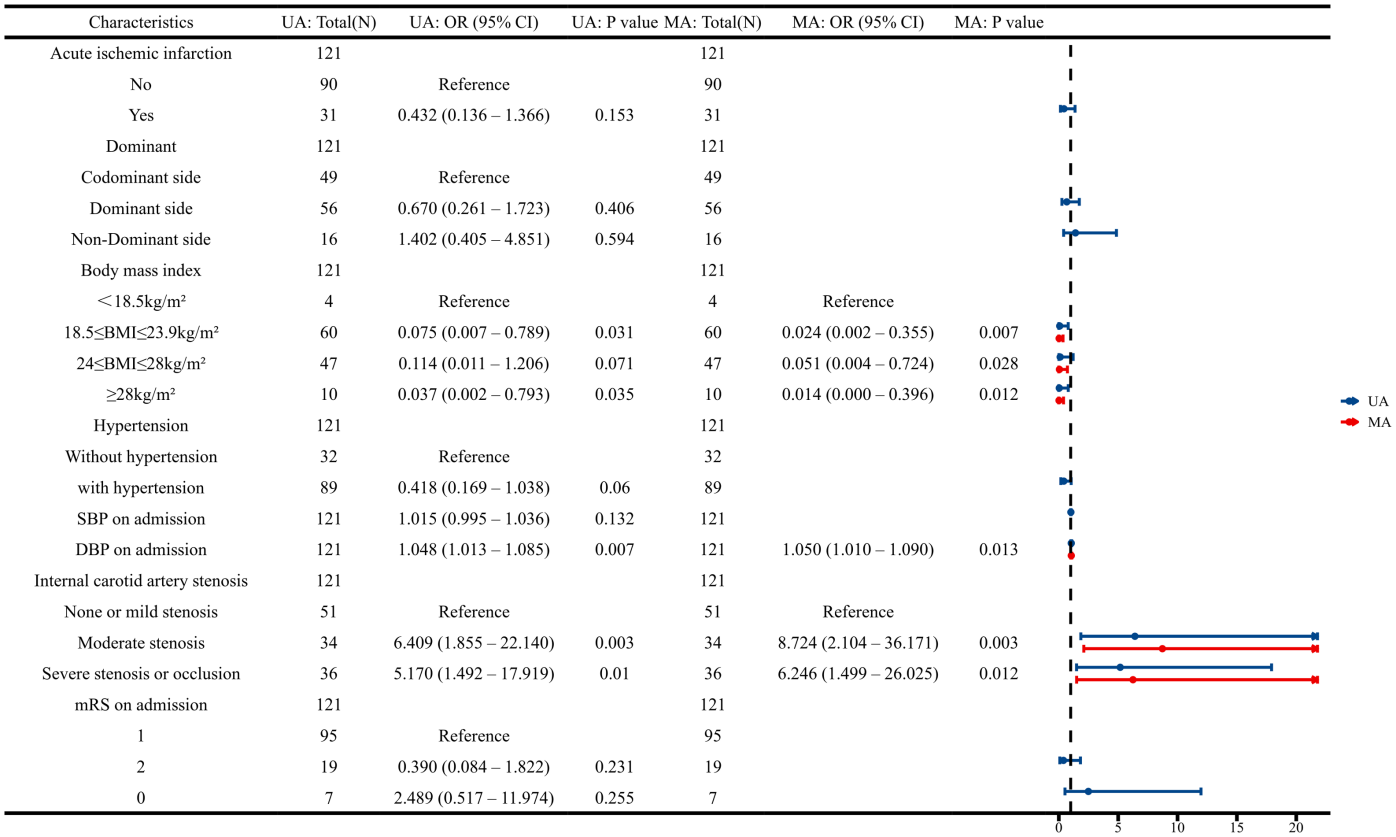
Multivariate logistic regression and forest plot for predicting bailout stenting. UA, univariate logistic regression analysis; MA, multivariate logistic regression analysis.

The results of the multivariate logistic regression analysis are presented in Figure 4. Elevated admission DBP and concomitant internal carotid artery stenosis were associated with an increased likelihood of conversion to bailout stenting, whereas higher BMI was identified as a protective factor.

**Figure 5:**
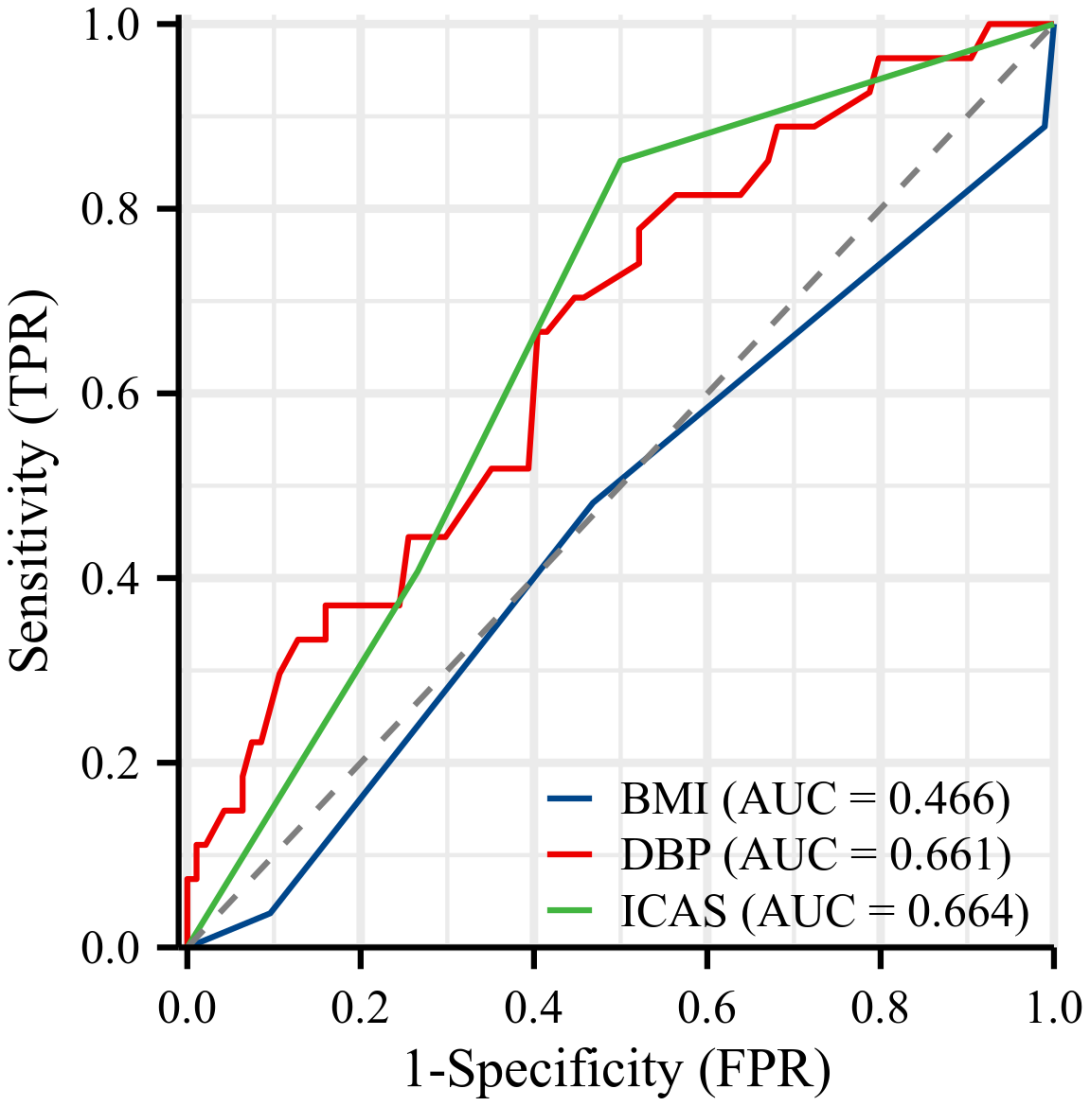
Receiver operating characteristic (ROC) curve for predicting the risk of bailout stenting. BMI, body mass index; DBP, diastolic blood pressure; ICAS, internal carotid artery stenosis.

The discriminative ability of each independent predictor was first evaluated using univariate ROC analysis (Figure 5). The area under the curve (AUC) for ICAS and DBP was 0.664 and 0.661, while BMI with an AUC of 0.466.

**Figure 6:**
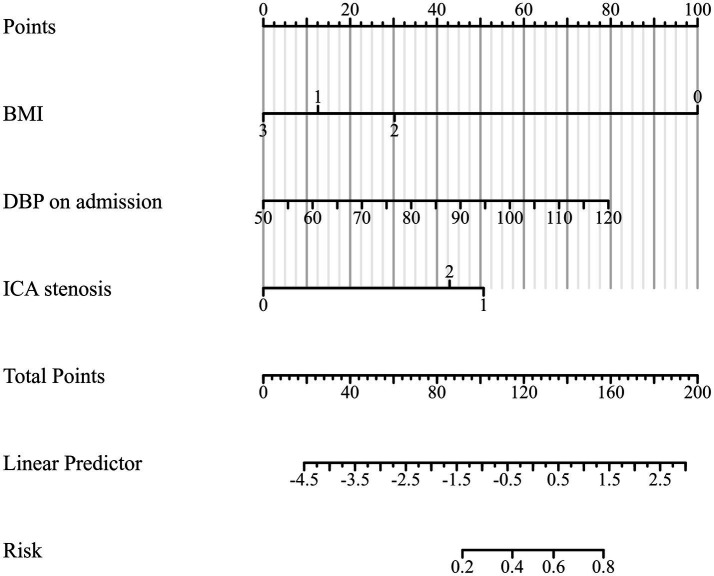
Nomogram for predicting the risk of bailout stenting. For an individual patient, a vertical line is drawn from each predictor according to the patient’s profile to the ‘Points’ axis to assign points for each variable. The points are then summed to obtain a total score. A vertical line from the total score is drawn to the ‘Linear Predictor’ axis for model calculation, and then extended to the ‘Risk’ axis to estimate the probability of BS. BMI, body mass index; DBP, diastolic blood pressure; ICA, internal carotid artery.

**Figure 7:**
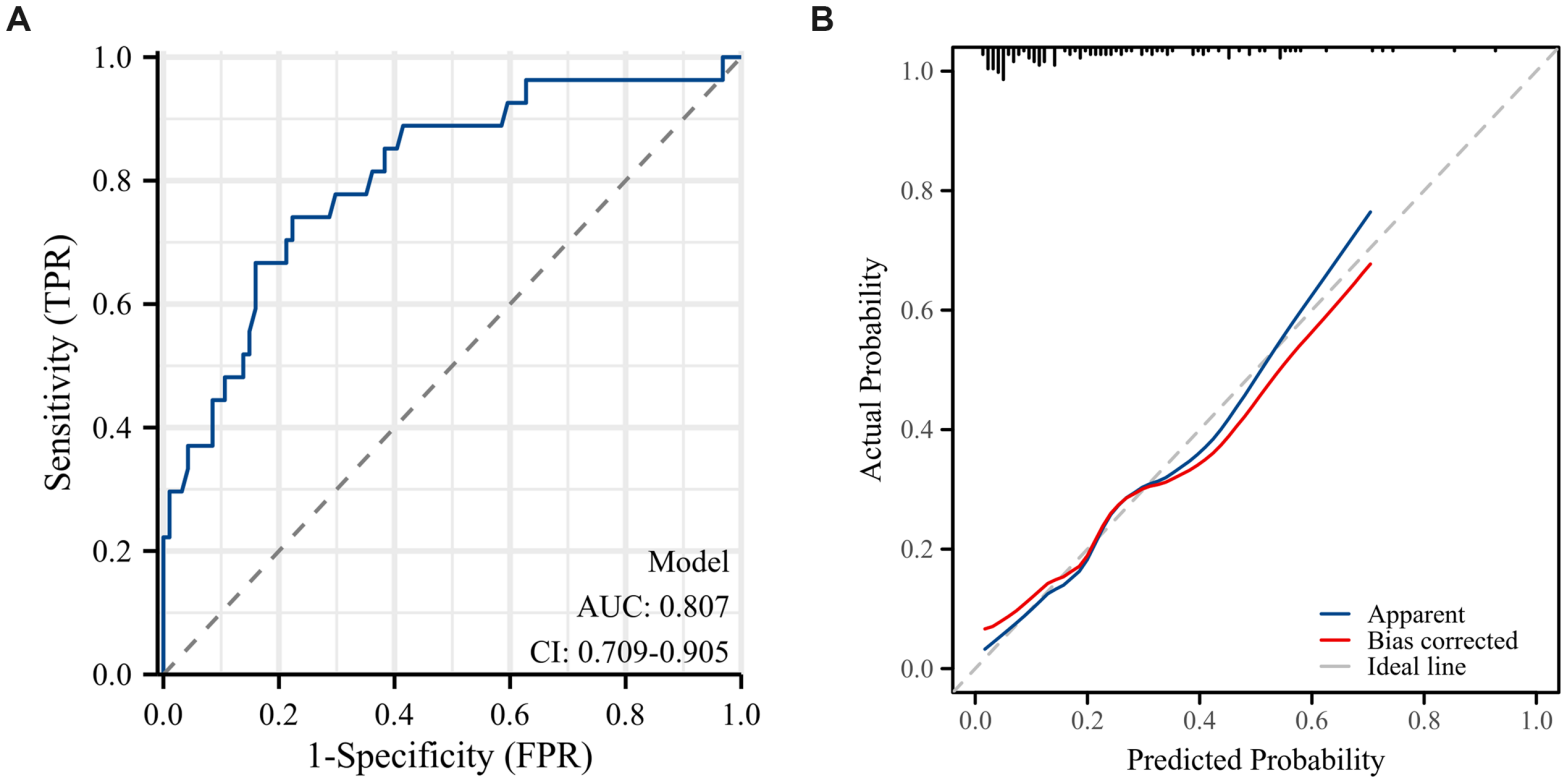
Performance of the predictive model. **(A)** Receiver operating characteristic (ROC) curve of the nomogram model. **(B)** Calibration curve for the risk prediction model of bailout stenting.

Based on the multivariable regression results, a visual nomogram was constructed to provide a practical tool for estimating the individualized probability of bailout stenting (Figure 6). Each independent predictor (BMI, DBP, and ICAS) was assigned a weighted score according to its regression coefficient, allowing for a comprehensive assessment of risk by summing the total points for each patient. The model exhibited robust discriminative power, with a concordance index (C-index) of 0.807 (95% CI: 0.709–0.906) (Figure 7A). Furthermore, the Hosmer–Lemeshow test (*p* = 0.9158) (Figure 7B) indicated no significant lack of fit, reflecting satisfactory calibration between the predicted and observed probabilities. This tool may facilitate pre-procedural risk stratification and individualized decision-making.

## 4 Discussion

In this single-center retrospective cohort of patients with symptomatic vertebral artery origin stenosis (VAOS), we evaluated the feasibility, safety, and angiographic outcomes of a drug-coated balloon (DCB)-based endovascular strategy in real-world practice. The main findings can be summarized as follows. First, DCB angioplasty demonstrated high procedural feasibility, with the majority of lesions successfully treated using a DCB-first approach. Second, ≥3-month angiographic follow-up demonstrated a relatively low rate of restenosis following DCB treatment. Third, bailout stenting (BS) was required in a subset of cases, primarily due to intraoperative dissection or significant elastic recoil; however, ≥12-month angiographic follow-up revealed no significant difference in restenosis rates between patients treated with DCB alone and those requiring BS. Finally, several clinical factors were identified as predictors of bailout stenting, and a preliminary nomogram was developed to facilitate individualized risk estimation. Taken together, these findings provide real-world evidence supporting the feasibility and potential clinical utility of a DCB-first strategy for symptomatic VAOS.

### 4.1 DCB in VAOS: rationale and evolving evidence

The vertebral artery origin represents a particularly challenging anatomical substrate for endovascular therapy. It is exposed to high pulsatile shear stress, frequent calcification, eccentric plaque morphology, and elastic recoil related to its proximity to the subclavian artery and surrounding musculoskeletal structures (23). These features partly explain the historically high restenosis rates observed after bare-metal stenting and conventional balloon angioplasty, with reported rates ranging from 20 to 50% within the first year (24–26). Drug-eluting stents were introduced to mitigate neointimal hyperplasia but have not consistently achieved durable patency in this vascular segment, and permanent implants remain susceptible to fracture, malapposition, and chronic inflammatory responses (24).

DCB angioplasty offers a conceptually attractive alternative by delivering antiproliferative drug locally while avoiding permanent scaffolding (25). In coronary and peripheral vascular beds, DCBs have demonstrated durable efficacy in reducing restenosis without increasing thrombosis or late adverse events (27–29). Translation of this strategy to extracranial cerebrovascular disease has been gradual, but accumulating evidence suggests similar mechanistic benefits. Early pilot studies and small observational cohorts demonstrated technical feasibility and favorable angiographic durability of DCB angioplasty in VAOS (18–22). More recently, the multicenter randomized DCB-VAOS trial provided the first high-level evidence that DCB significantly reduces 12-month restenosis compared with bare-metal stents while maintaining comparable periprocedural and 1-year safety outcomes (23).

The present study complements this emerging evidence base by providing real-world data from one of the largest reported DCB-treated VAOS cohorts to date. While the randomized trial established efficacy under controlled conditions, our cohort reflects routine clinical practice, including patients with more complex anatomy, concomitant atherosclerotic disease, and intraoperative complications requiring procedural adaptation. Together, these data suggest that DCB angioplasty represents a viable revascularization strategy for VAOS across diverse clinical settings.

It should be noted that, unlike coronary DCB interventions where systematic lesion preparation is widely recommended, there is currently no standardized procedural consensus regarding optimal lesion preparation strategies in VAOS interventions (26). In our study, predilatation was not routinely performed and was reserved primarily for cases of device-crossing failure. This conservative approach was adopted to minimize mechanical injury to the vertebral artery ostium, a vascular segment characterized by complex hemodynamics, frequent eccentric calcification, and marked tortuosity. Excessive mechanical manipulation in this region may increase the risk of severe dissection or vessel rupture. The resulting intimal injury can trigger an inflammatory cascade, thereby promoting restenosis (24). Nevertheless, we acknowledge that the absence of systematic lesion preparation may potentially affect drug transfer efficiency and acute lumen optimization. Therefore, this strategy represents a potential methodological limitation of the present study and warrants further investigation in future prospective studies aimed at defining optimal procedural protocols for DCB-based VAOS revascularization.

### 4.2 Procedural safety, angiographic durability and clinical outcomes

In addition to angiographic outcomes, the present study also demonstrated a favorable safety profile for DCB angioplasty. No major perioperative complications—including death, major stroke, or vessel rupture—were observed. Minor procedural complications were infrequent and were successfully managed during the intervention. These findings suggest that DCB angioplasty is a safe therapeutic option for the treatment of symptomatic VAOS in routine clinical practice.

In our cohort, DCB angioplasty demonstrated favorable mid-term angiographic outcomes. Among patients with ≥3-month imaging follow-up, the restenosis rate in the DCB group was significantly lower than that in the BS group. Sensitivity analyses addressing missing imaging follow-up yielded consistent results, supporting the robustness of this observation despite incomplete follow-up data.

However, among patients with imaging follow-up beyond 12 months, the difference in restenosis rates between the DCB and BS groups was no longer statistically significant. This finding should be interpreted with caution, as the number of patients with long-term imaging follow-up was relatively limited and no sensitivity analysis was performed for this subgroup. Importantly, these findings suggest that the requirement for bailout stenting does not necessarily compromise long-term vessel patency.

These results have practical implications for procedural strategy. While DCB angioplasty may achieve favorable mid-term outcomes in many patients, the use of selective bailout stenting appears to maintain acceptable long-term angiographic results when vessel compromise occurs during intervention. These findings reinforce the concept that selective stent rescue does not negate the longer-term benefits of DCB-based therapy.

Nevertheless, angiographic restenosis remains a surrogate endpoint, and its relationship with recurrent posterior circulation ischemic events is not fully defined. Prior studies in VAOS have shown discordance between restenosis severity and clinical recurrence, particularly when adequate collateral circulation exists (22).

In the DCB-VAOS randomized trial, despite a marked reduction in angiographic restenosis, clinical event rates—including stroke, TIA, and death—were low and did not differ significantly between treatment arms (23). Similarly, clinical events in our cohort were infrequent, limiting meaningful comparative analysis. These observations suggest that while angiographic durability is an important marker of procedural success, future studies should prioritize patient-centered outcomes, including recurrent ischemic events, functional status, cognitive trajectories, and health-related quality of life.

### 4.3 Bailout stenting: a part of a DCB-first strategy

In our cohort, BS was required in approximately 22% of patients, a rate consistent with prior observational reports of DCB angioplasty in extracranial cerebrovascular lesions (18–21) and broadly comparable to crossover or rescue stenting rates in peripheral vascular DCB studies (27, 30, 31). The predominant trigger for BS was flow-limiting dissection, elastic recoil and suboptimal residual lumen gain also contributed in selected cases.

Importantly, patients requiring BS demonstrated angiographic outcomes comparable to those treated with balloon-only strategies. This finding is consistent with evidence from coronary artery studies, indicating that bailout stenting yields similar long-term safety and efficacy outcomes compared to successful DCB alone or primary stenting (25, 30). These findings suggest that DCB-first with selective stent rescue—may preserve the antiproliferative benefit of drug delivery while mitigating the risks of acute vessel compromise. From a procedural standpoint, this strategy offers operators flexibility, enabling tailored escalation based on lesion response rather than committing upfront to permanent implantation.

### 4.4 Predictors of bailout stenting: preliminary clinical considerations

An additional exploratory finding of this study is the identification of factors independently associated with the need for BS, including elevated admission diastolic blood pressure, and concomitant carotid artery stenosis, while higher body mass index appeared protective.

These associations may reflect underlying differences in vascular remodeling and systemic atherosclerotic burden. Elevated diastolic blood pressure may increase arterial wall stress and reduce vascular compliance, thereby predisposing lesions to dissection during balloon dilation. Similarly, the presence of ipsilateral carotid artery stenosis may indicate more diffuse atherosclerotic disease and impaired vascular elasticity.

Interestingly, higher BMI appeared to be associated with a lower risk of BS in our analysis. However, the discriminative ability of BMI alone was limited, suggesting that its predictive value is modest. Therefore, this observation should be interpreted cautiously.

The predictors of BS identified in our study differ from those reported in coronary and peripheral vascular interventions (27–29, 31). This discrepancy largely stems from the absence of routine intravascular ultrasound (IVUS) in our real-world practice; consequently, our predictive model is intrinsically weighted toward systemic hemodynamic and macro-clinical variables (e.g., DBP, concurrent ICA stenosis) rather than micro-anatomical lesion characteristics (e.g., calcification arc, lipid core burden, and precise vessel angulation). While this limitation precludes a granular biomechanical analysis of the plaque, our findings nevertheless provide a pragmatic risk stratification tool based on readily accessible clinical data. Given the moderate sample size, these results should be interpreted cautiously as a foundational clinical framework. Future prospective studies integrating high-resolution intravascular imaging are essential to construct multidimensional models that couple systemic hemodynamic profiles with local plaque biomechanics.

The nomogram derived from these predictors demonstrated acceptable discrimination and calibration in internal validation, suggesting potential utility for procedural risk stratification. However, given the exploratory nature of the model and the modest sample size, external validation in independent cohorts is essential before clinical implementation.

### 4.5 Implications for clinical practice

Taken together, these findings support a pragmatic revascularization paradigm for VAOS: initial DCB angioplasty with selective bailout stenting when necessary. This approach leverages the antiproliferative benefits of drug delivery while preserving procedural flexibility and avoiding routine permanent implantation in a mechanically hostile vascular segment. From a clinical standpoint, identification of patients at higher risk for BS—such as those with elevated diastolic blood pressure or concomitant carotid disease—may facilitate procedural planning, device selection, and patient counseling.

Furthermore, our data suggest that the need for BS should not be interpreted as procedural failure but rather as an adaptive component of a stepwise endovascular strategy. In experienced centers, this hybrid approach may offer a favorable balance between safety and durability, particularly in patients unsuitable for surgical revascularization or refractory to medical therapy.

### 4.6 Study limitations

Several limitations should be acknowledged. First, the retrospective, single-center design introduces potential selection bias and limits generalizability. Second, follow-up imaging was incomplete, which may have affected restenosis estimates. In real-world practice, asymptomatic patients frequently declined repeat invasive or advanced imaging. However, sensitivity analyses comparing patients with and without follow-up imaging did not reveal significant baseline differences. Third, the absence of a contemporaneous control group precludes direct comparison with alternative revascularization strategies, including primary stenting or optimized medical therapy. Fourth, a notable divergence in our protocol from established coronary DCB consensus is the omission of routine pre-dilation. While this “DCB-first” strategy without systematic lesion preparation was adopted to minimize mechanical trauma to the fragile vertebral ostium, inadequate plaque modification prior to drug delivery may have compromised paclitaxel absorption and potentially contributed to the observed need for bailout stenting. Fifth, due to the lack of approval for cerebrovascular IVUS at our center during the study period, we could not assess plaque characteristics (e.g., calcification burden, fibrous cap thickness). In addition, the relatively small number of events may have limited the stability of the multivariable model. Consequently, the impact of plaque morphology on DCB expansion and long-term durability remains unknown and warrants further investigation. Finally, the study was not prospectively powered to detect differences in clinical outcomes, limiting inference regarding stroke prevention.

## 5 Conclusion

DCB angioplasty was associated with favorable angiographic durability in symptomatic VAOS, with similar long-term patency between balloon-only and bailout stent strategies. Higher diastolic blood pressure, and concomitant carotid stenosis were associated with bailout stenting. These findings support a DCB-first approach with selective stent rescue and warrant prospective validation.

## Data Availability

The raw data supporting the conclusions of this article will be made available by the authors, without undue reservation.
