# Supplementary material for: Drug-coated balloon angioplasty for symptomatic vertebral artery origin stenosis: a real-world analysis of efficacy and predictors for bailout stenting
Source: Front Neurol. 2026 Apr 14;17:1804766. doi: 10.3389/fneur.2026.1804766 (PMC13120963; doi:10.3389/fneur.2026.1804766)
Supplement: Supplementary file 1 [file Supplementary_file_1.docx]

Supplementary Material

**Supplementary Table S1：**Comparison of baseline characteristics between patients with and without imaging follow-up at ≥-3 months and ≥12 months

| Variables | Total  (n= 121) | ≥3-month follow-up  （n=61） | No imaging follow-up within 3 months  (n=60) | *P* Value | ≥12-month follow-up  (n=35) | No imaging follow-up ≥12 months(n=86) | | *P* Value |
| --- | --- | --- | --- | --- | --- | --- | --- | --- |
| Age, year (M, IQR) | 70.00(61.00, 75.00) | 71.00 (61.00, 75.00) | 67.50 (61.00, 75.00) | 0.607 ^*^ | 72.00 (64.50, 75.00) | | 67.50 (58.75, 74.00) | 0.101 ^*^ |
| Male, n (%) | 99 (81.82) | 55 (90.16) | 44 (73.33) | 0.016^†a^ | 32 (91.43) | | 67 (77.91) | 0.080 ^†^ |
| BMI, Kg/m² (m ± s) | 24.09 ± 3.08 | 24.19 ± 2.76 | 23.98 ± 3.39 | 0.700^‡^ | 24.34 ± 2.82 | | 23.99 ± 3.19 | 0.573^‡^ |
| Admission SBP, mmHg (m ± s) | 141.16 ± 21.70 | 143.39 ± 21.42 | 138.88 ± 21.93 | 0.255^‡^ | 143.49 ± 19.49 | | 140.21 ± 22.58 | 0.454^‡^ |
| Admission DBP, mmHg (m ± s) | 82.00 ± 13.19 | 83.25 ± 12.46 | 80.73 ± 13.88 | 0.297^‡^ | 83.57 ± 12.03 | | 81.36 ± 13.65 | 0.405^‡^ |
| Fasting blood glucose, mmol/L, (M, IQR) | 5.70 (5.00, 7.20) | 5.60 (4.90, 7.40) | 5.78 (5.14, 7.08) | 0.452^*^ | 5.70 (4.74, 6.38) | | 5.69 (5.00, 7.27) | 0.558^*^ |
| Triglycerides level, mmol/L (M, IQR) | 1.27 (0.92, 1.85) | 1.19 (0.94, 1.86) | 1.35 (0.87, 1.82) | 0.860^*^ | 1.27 (0.99, 1.92) | | 1.27 (0.86, 1.80) | 0.464^*^ |
| Total cholesterol, mmol/L (M, IQR) | 3.61 (2.98, 4.28) | 3.52 (2.92, 4.15) | 3.71 (3.13, 4.30) | 0.300^*^ | 3.52 (2.86, 4.24) | | 3.66 (3.07, 4.28) | 0.334^*^ |
| LDL cholesterol level，mmol/L (M,IQR） | 2.31 (1.85, 2.88) | 2.23 (1.84, 2.83) | 2.44 (1.91, 2.94) | 0.262^*^ | 2.15 (1.75, 2.84) | | 2.37 (1.92, 2.88) | 0.376^*^ |
| HDL cholesterol level， mmol/L （M,IQR） | 1.01 (0.87, 1.18) | 1.01 (0.88, 1.12) | 1.02 (0.87, 1.22) | 0.502^*^ | 0.97 (0.86, 1.10) | | 1.04 (0.91, 1.22) | 0.103^*^ |
| Hypertension, n (%) | 89 (73.55) | 44 (72.13) | 45 (75.00) | 0.721^†^ | 27 (77.14) | | 62 (72.09) | 0.568^†^ |
| Diabetes mellitus, n (%) | 48 (39.67) | 21 (34.43) | 27 (45.00) | 0.235^†^ | 12 (34.29) | | 36 (41.86) | 0.440^†^ |
| Hyperlipidemia, n (%) | 9 (7.44) | 4 (6.56) | 5 (8.33) | 0.979^†^ | 3 (8.57) | | 6 (6.98) | 1.000^†^ |
| Cardiopathy, n (%) | 28 (23.14) | 10 (16.39) | 18 (30.00) | 0.076^†^ | 5 (14.29) | | 23 (26.74) | 0.141^†^ |
| Atrial fibrillation, n (%) | 3 (2.48) | 0 (0.00) | 3 (5.00) | 0.236^§^ | 3 (5.00) | | 0 (0.00) | 0.556^§^ |
| Current smoking, n (%) | 17 (14.05) | 10 (16.39) | 7 (11.67) | 0.454^†^ | 3 (8.57) | | 14 (16.28) | 0.413^†^ |
| Alcohol consumption, n (%) | 7 (5.79) | 3 (4.92) | 4 (6.67) | 0.982^†^ | 2 (5.71) | | 5 (5.81) | 1.000^†^ |
| Preoperative Stenosis percentage, % (M, IQR) | 0.83 (0.75, 0.90) | 0.83 (0.78, 0.90) | 0.82 (0.75, 0.91) | 0.846^*^ | 0.83 (0.76, 0.91) | | 0.83 (0.75, 0.90) | 0.936^*^ |
| Target vessel side, n (%) |  |  |  | 0.939^†^ |  | |  | 0.065 ^†^ |
| Right | 71 (58.68) | 36 (59.02) | 35 (58.33) |  | 16 (45.71) | | 55 (63.95) |  |
| Left | 50 (41.32) | 25 (40.98) | 25 (41.67) |  | 19 (54.29) | | 31 (36.05) |  |
| Dominant, n (%) |  |  |  | 0.884^†^ |  | |  | 0.976^†^ |
| Codominant side | 49 (40.50) | 26 (42.62) | 23 (38.33) |  | 14 (40.00) | | 35 (40.70) |  |
| Dominant side | 56 (46.28) | 27 (44.26) | 29 (48.33) |  | 16 (45.71) | | 40 (46.51) |  |
| Non-Dominant side | 16 (13.22) | 8 (13.11) | 8 (13.33) |  | 5 (14.29) | | 11 (12.79) |  |
| Acute infarction on imaging, n (%) | 31 (25.62) | 16 (26.23) | 15 (25.00) | 0.877^†^ | 12 (34.29) | | 19 (22.09) | 0.164^†^ |
| Internal carotid artery stenosis, n (%) |  |  |  | 0.588^§^ |  | |  | 0.766^§^ |
| No | 51 (42.15) | 23 (37.70) | 28 (46.67) |  | 13 (37.14) | | 38 (44.19) |  |
| Mild or moderate | 34 (28.10) | 19 (31.15) | 15 (25.00) |  | 11 (31.43) | | 23 (26.74) |  |
| Severe or total occlusion | 36 (29.75) | 19 (31.15) | 17 (28.33) |  | 11 (31.43) | | 25 (29.07) |  |

M = median; IQR = interquartile range; DCB = drug-coated balloon; BS=bailout stenting; BMI = body mass index; SBP = systolic blood pressure; DBP = diastolic blood pressure; FBG = fasting blood glucose; LDL=low-density lipoprotein; HDL = high-density lipoprotein; ^a^:*P*＜0.05 *: Mann-Whitney test, †: Chi-square test, ‡:t-test, §: Fisher exact.
